# Crucial Roles of Two Hydrated Mg^2+^ Ions in Reaction Catalysis of the Pistol Ribozyme

**DOI:** 10.1002/anie.201912522

**Published:** 2020-01-09

**Authors:** Marianna Teplova, Christoph Falschlunger, Olga Krasheninina, Michaela Egger, Aiming Ren, Dinshaw J. Patel, Ronald Micura

**Affiliations:** ^1^ Institute of Organic Chemistry and Center for Molecular Biosciences Leopold-Franzens University Innrain 80–82 6020 Innsbruck Austria; ^2^ Structural Biology Program Memorial Sloan-Kettering Cancer Center New York New York 10065 USA; ^3^ Life Sciences Institute Zhejiang University Hangzhou Zhejiang 310058 China

**Keywords:** magnesium, oligonucleotides, reaction mechanisms, RNA, structure–function relationships

## Abstract

Pistol ribozymes constitute a new class of small self‐cleaving RNAs. Crystal structures have been solved, providing three‐dimensional snapshots along the reaction coordinate of pistol phosphodiester cleavage, corresponding to the pre‐catalytic state, a vanadate mimic of the transition state, and the product. The results led to the proposed underlying chemical mechanism. Importantly, a hydrated Mg^2+^ ion remains innersphere‐coordinated to N7 of G33 in all three states, and is consistent with its likely role as acid in general acid base catalysis (δ and β catalysis). Strikingly, the new structures shed light on a second hydrated Mg^2+^ ion that approaches the scissile phosphate from its binding site in the pre‐cleavage state to reach out for water‐mediated hydrogen bonding in the cyclophosphate product. The major role of the second Mg^2+^ ion appears to be the stabilization of product conformation. This study delivers a mechanistic understanding of ribozyme‐catalyzed backbone cleavage.

## Graphical abstract

**Role play**: Transition‐state mimic and product crystal structures suggest that two hydrated metal ions are involved in pistol ribozyme cleavage chemistry. One of them remains innersphere‐coordinated to N7 of a guanosine and plays a key role in catalysis.

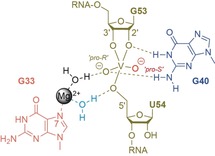

Small self‐cleaving ribozymes catalyze site‐specific cleavage of their own phosphodiester backbone. They are widely distributed in nature and are essential for rolling‐circle‐based replication of satellite and pathogenic RNAs.^1^, ^2^, ^3^, ^4^, ^5^, ^6^, ^7^, ^8^, ^9^, ^10^, ^11^, ^12^, ^13^ Comparative genomic analysis led to the discovery of novel self‐cleaving ribozymes, named twister, twister‐sister, pistol, and hatchet.^14^, ^15^ For the first three classes, the three‐dimensional architectures^16^, ^17^, ^18^, ^19^, ^20^, ^21^, ^22^, ^23^, ^24^, ^25^ in pre‐cleavage states were solved by X‐ray crystallography, and very recently, also the first structure of a hatchet ribozyme (product) has been determined.^26^ These structures represent a thorough basis to explore the chemical mechanism of the site‐specific transesterification reactions^6^, ^27^ that are catalyzed by these ribozymes (Figure 1 a). The assessment of ribozyme X‐ray structures is demanding because these molecules require structural flexibility at the cleavage site and active‐site pocket for the spatio‐temporal correlation to enable the chemical reaction. Therefore, careful structure–function analysis in solution by targeted mutagenesis is necessary. Complementarily, a potent approach to advance our understanding of ribozyme catalysis is the structure elucidation of transition‐state (TS) analogues. This approach is, however, a challenging experimental task. Suitable mimics for the pentavalent TS of a phosphorane are rare and those of vanadate analogues have to date been solved for hairpin and hammerhead ribozymes only.^28^, ^29^, ^30^

**Figure 1:**
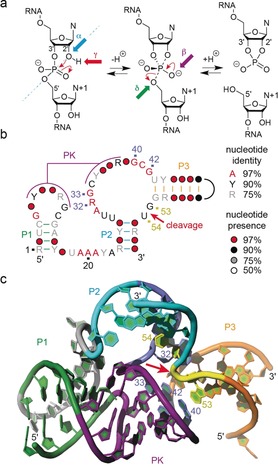
Self‐cleaving ribozymes. a) Model for phosphodiester cleavage:^6^, ^27^ The internucleotide linkage (“scissile” phosphate) passes a pentacoordinate transition state that gives two cleavage products carrying a 2′,3′‐cyclic phosphate terminus and a 5′‐hydroxy terminus: α, in‐line nucleophilic attack, *S*_N_2‐like (blue); β, neutralization of the (developing) negative charge on nonbridging phosphate oxygen atoms (purple); γ, deprotonation of the 2′‐hydroxy group (red); and δ, neutralization of negative charge on the 5′‐oxygen atom by protonation (green). b) Consensus sequence and secondary structure model for pistol ribozymes;^15^ pseudoknot (PK); black line indicates variable loop; nucleotide numbers refer to panel below. c) Crystal structure of the precatalytic fold of the dG53‐modified ribozyme (pdb code 5K7C).^24^ The cleavage site G53 U54 (yellow) and the closest residues A32, G33, G40, and G42 (marine blue) are numbered; red arrow indicates the scissile phosphate.

In the present study we set out to obtain conformational snapshots along the reaction coordinate of the pistol ribozyme phosphordiester cleavage (Figure 1). We succeeded in solving the X‐ray structures of both its TS analogue vanadate (at 2.8 Å resolution) and the ternary 2′,3′ cyclophosphate product complex (at 2.65 Å resolution; Figure 2; see Figure S1 in the Suppporting Information for stereoviews). Together with our previously obtained structure of the pre‐cleavage conformation of the pistol ribozyme at 2.7 Å resolution^24^ (Figures 1 c and 2 a), we achieved an architectonic framework for the cleavage reaction, allowing profound proposal for the underlying chemical mechanism. In short, a divalent ion (Mg^2+^, Mn^2+^) strictly remains innersphere‐coordinated to N7 of G33 in all three states (pre‐cleavage,^24^, ^31^ TS analogue, and post‐cleavage) and allows simultaneous water‐mediated interaction with the *pro‐R* nonbridging oxygen atom of the scissile phosphate. Strikingly, a second hydrated Mg^2+^ ion moves from its binding pocket in the pre‐cleavage state towards the scissile phosphate in the TS and becomes coordinated between the *pro‐S* oxygen atom and the N7 of A38 in the product. Our findings suggest the potential participation of a hydrated Mg^2+^ as a general acid for proton transfer to the 5′O leaving group (δ catalysis) in pistol ribozyme cleavage, and also suggest an additional role for a second Mg^2+^ in the conformational stabilization of the product.

**Figure 2:**
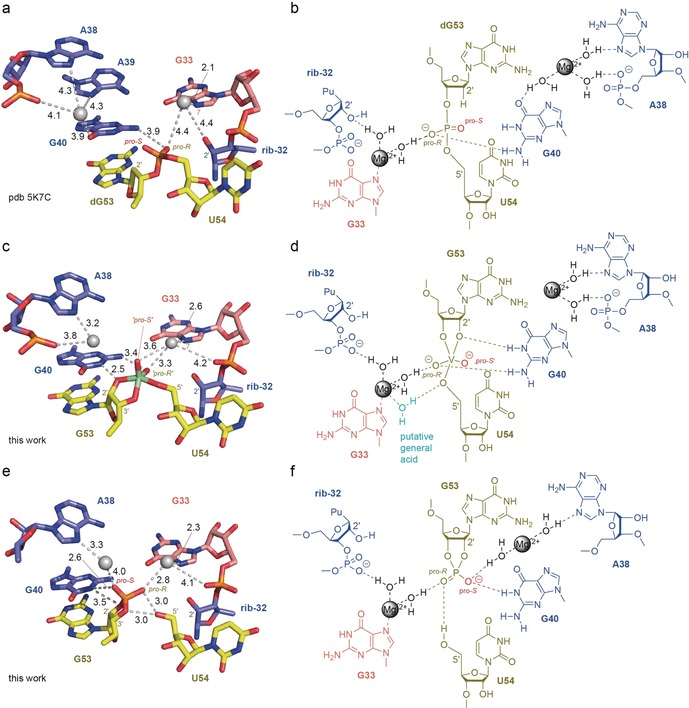
Crystal structures and structural formulas with implicated mechanistic aspects for pistol ribozyme pre‐cleavage (a,b), TS analogue (c,d), and post‐cleavage (e,f) conformations. Crucial atom distances (below 4.5 Å) that indicate direct or water‐mediated hydrogen bonds and/or metal ion interactions are illustrated by dashed lines. The values in black represent distances in Å. Note that the number of indicated distances can exceed the number of possible hydrogen bonds for a particular atom. For discussion see the main text. For stereo views see Figure S1.

Most X‐ray structures of small self‐cleaving ribozymes refer to the precatalytic fold, with the nucleoside 5′ of the scissile phosphate substituted by the corresponding 2′‐deoxynucleoside to prevent cleavage. This arrangement was also the case for our recently solved structure of the pistol ribozyme (Figure 2 a).^24^ Modelling of a hydroxy group onto the 2′‐deoxyribose revealed an “in‐line” orientation of this 2′O, ready for attack at the phosphorus atom of the “to‐be‐cleaved” P−O5′ bond, which is in accordance with mechanistic requirements (S_N_2‐like). Furthermore, evaluation of the structure by atomic mutagenesis experiments pointed to the crucial role of a divalent cation that was innersphere‐coordinated to N7 of an active site guanine (G33).^31^ The assignment of a metal ion coordinated to N7 of purine nucleobases can be ambiguous at a resolution of 2.7 Å,^32^ and hence, efforts were made to verify the metal ion coordination by Mn^2+^ soaking. The obtained anomalous electron‐density map and the coordination distance of 2.1 Å were supportive for a divalent metal binding site.^24^ Most importantly, our finding that deletion of the coordination site (mutation of G33 to 7‐deazaG33) rendered the ribozyme almost completely inactive, even in the presence of high concentrations of Mg^2+^ ions, suggested an important role of Mg^2+^ in catalysis.^31^ We note here that the distance of the N7‐coordinated Mg^2+^ to the *pro‐R* oxygen atom of the scissile phosphate was 4.4 Å and is consistent with a water‐mediated hydrogen‐bond interaction as indicated in Figures 2 a,b.

To increase our understanding of the pistol ribozyme's catalytic mechanism, we have now determined its structure when complexed with a TS mimic at 2.8 Å resolution. To obtain the crystal structure of the TS analogue, we mixed the three RNA strands shown in Figure S2. The resulting complex lacks the scissile phosphate but retains the 2′‐, 3′‐, and 5′‐hydroxy groups at the cleavage site. This complex was co‐crystallized with NH_4_VO_3_. Details are provided in the Supporting Information (see Tables S1 and S2). The electron density in the active site can be assigned to vanadate based on its size, shape, and anomalous scattering (Figure 3). Distances between the 2′‐, 3′‐, and 5′‐oxygen atoms and the vanadium atom are consistent with direct coordination between the oxygen atoms and the vanadium atom.

**Figure 3:**
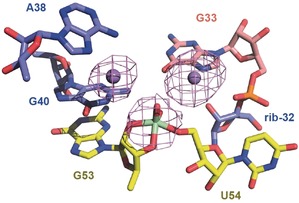
View of the active site of the pistol ribozyme vanadate complex (transition state analog). Crystals of the ribozyme with a vanadate linkage replacing the scissile phosphate were grown in Mg^2+^ containing buffer and then soaked into a cryo‐stabilization buffer containing Mn^2+^. An anomalous diffraction map, contoured here at 3 σ, was calculated to determine the positions of the Mn^2+^ ions and vanadate bound to the ribozyme.

The structure of the TS analogue reveals fine but significant rearrangements relative to the pre‐cleavage structure (Figure 2 c,d). Most importantly, while the key players (ribose‐32, G33, G40, G53, U54) in the active site only minimally alter their positions, the conformation of the backbone of the cleavage site is changed. The nonbridging oxygen centers of the scissile phosphate are rotated, bringing both the *pro‐S* oxygen and *pro‐R* oxygen centers much closer to the Hoogsteen face of G33 (shifting from 6.2 to 3.6 Å and from 4.4 to 3.3 Å towards N7‐G33, respectively; Figure 2 a,c). As a result, the divalent metal ion (whose innersphere coordination to N7‐G33 was verified by the anomalous signal in the structure of Mn^2+^ soaked crystals; Figure 3) potentially interacts through water‐mediated hydrogen bonding with the nonbridging oxygen atoms of the scissile phosphate. This ion is also at a 4 Å distance to the 5′O leaving group of U54 such that it could position a water molecule to serve as a general acid, in the cleavage reaction, for proton transfer to stabilize the leaving 5′O (Figure 2 c,d).

Furthermore, our TS analogue structure strongly suggests the bidentate interaction of G40 with the phosphorane TS during cleavage. In the vanadate complex both, N1 of G40 and N2 of G40 are within hydrogen‐bonding distance to the 2′O (2.5 Å) and the *pro‐R* oxygen atom (3.4 Å), respectively (Figure 2 c,d). A direct role of G40 as a base in general acid‐base catalysis is widely accepted according to functional mutagenesis assays,^24^, ^31^, ^33^ and it is now complemented and consistent with the structural framework provided here. We also note that the cleavage rate differences observed for phosphorothioate substrates are consistent with our structures.^33^, ^34^ The significantly reduced rate measured for the *R*_P_ diastereomer is in agreement with a direct recognition of the phosphorothioate moiety by G40. Furthermore, because the activity was not restored by addition of Mn^2+^ ions,^35^, ^36^ water‐mediated (outersphere) rather than innersphere coordination between the phosphorothioate and the metal ion is likely and the M^2+^‐′*proR*‐O′ distance of 3.3 Å in our TS structure supports this notion further. In contrast to the *R*_P_ diastereomer, cleavage of the *S*_P_ diastereomer was only minimally diminished and this finding is also plausible because the *pro‐S* oxygen atom has no direct interactions in our structures of the TS analogue and the pre‐cleavage state.

The third structural snapshot on the pistol ribozyme reaction coordinate that we have solved represents the post‐cleavage state (Figure 2 e,f). In this product structure, G53 and U54 are slightly more distant to each other compared to the TS analogue. Moreover, the ribose moiety of G53 finds itself in a distinctly different orientation, with its 2′,3′‐cyclic phosphate rotated relative to the vanadate moiety in the TS mimic. As a result, the cyclic phosphate is recognized by a 2.6 Å hydrogen bond between G40‐N1 and its *pro‐S* nonbridging oxygen atom, and a 3.0 Å hydrogen bond between the free 5′OH and its nonbridging *pro‐R* oxygen atom. Notably, the divalent metal ion remains coordinated to the N7 of G33 (2.3 Å) and its 2.8 Å distance to the *pro‐R* oxygen of the cyclic phosphate offers the possibility for a water‐mediated interaction.

Importantly, the structure of the pistol product complex clearly shows a second metal ion that was located at a 4.0 Å distance to the *pro‐S* oxygen atom of the cyclic phosphate, and therefore also offers the possibility for outershell, water‐mediated hydrogen bonding. A second divalent metal ion was also present in the TS analogue and the pre‐cleavage structures, however, with greater distances of 4.8 Å and 6.3 Å, respectively. Consequently, this ion and the scissile phosphate approached each other along the reaction coordinate as illustrated in the superposition of the three structures in Figure 4 and Figure S3.

**Figure 4:**
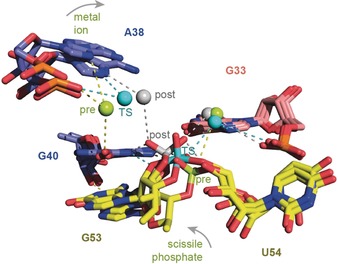
Superpositions of the pre‐cleavage state (M^2+^ and scissile phosphate in green color), TS analogue vanadate (cyan), and post‐cleavage state (grey), illustrating the movement of a Mg^2+^ ion in proper distance for water‐mediated (outershell) hydrogen‐bond interactions with O6 of G40 in the pre‐cleavage state and the *pro‐S* nonbonding oxygen atom of the scissile phosphate in the post‐cleavage state, respectively. For discussion see main text.

According to the new product and TS analogue structures, and the formerly solved precatalytic pistol ribozyme structure, possible (outershell) coordination sites for this second hydrated metal ion are A38 (N7 and phosphate; highly conserved as purine) and A39 (N7; nucleotide identity not conserved; Figures 2 a,c,e). We therefore analyzed whether deletion of the N7 coordination sites, by replacement with 7‐deazaadenosine (c^7^A; atomic mutagenesis), has an impact on ribozyme activity (Figure 5). By applying a previously established fluorescence spectroscopic assay (based on A57Ap substrate labeling),^24^, ^31^ cleavage kinetics were determined. The cleavage rate was comparable to the wildtype for the A38c^7^A mutant, 1.5‐fold reduced for the A39c^7^A mutant, and 8‐fold reduced for the A38c^7^A‐A39c^7^A double mutant (Figures 5 c–f and Table 1).

**Figure 5:**
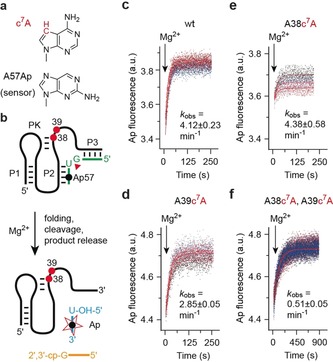
Atomic mutagenesis of potential coordination sites in the second metal binding site in *env25* pistol ribozyme to analyze the impact on self‐cleavage. a) Chemical structures of c7A and 2‐aminopurine. b) Design of the fluorescence‐based assay to monitor self‐cleavage in real time. c) Fluorescence time courses of wildtype ribozyme for rate determination; MgCl_2_ was added at *t*=0. Conditions: c(RNA)=0.5 μm (1:1 ratio), 50 mm KMOPS, 100 mm KCl, 23 °C, pH 7.5; mixing was performed with a stopped‐flow apparatus resulting in 10 mm Mg^2+^ concentration (*k*_obs_ obtained from three independent experiments). a.u.=arbitrary units. d) Same as (c) but for A39c7A mutant. e) Same as (c) but for A38c7A mutant. f) Same as (c) but for A38c7A‐A39c7A double mutant.

**Table 1:** Cleavage rates of the *env25* pistol ribozyme and mutants.

Ribozyme variant^[a]^	*k*_obs_ [min^−1^]
wildtype	4.12±0.23^[a]^
A38c^7^A	4.38±0.58^[a]^
A39c^7^A	2.85±0.05^[a]^
A38c^7^A, A39c^7^A	0.51±0.05^[a]^
G40Ap	4.21±0.15^[b]^
G40I	0.018±0.002^[c]^
G33c^7^G	0.0043±0.0004^31^

[a] Fluorescence assay (A57Ap), conditions: *c*(RNA)=0.5 μm each strand (1:1 ratio), 50 mm KMOPS, 100 mm KCl, 10 mm Mg^2+^, 23 °C, pH 7.5. [b] Same as a, but A40Ap. [c] HPLC assay, conditions: *c*(RNA)=55 μm each strand (1:1 ratio), 30 mm HEPES, 100 mm KCl, 2 mm Mg^2+^, 23 °C, pH 7.5.

Alternative assays using HPLC analysis independently confirmed this behavior (see Figures S4 and S5). Clearly, the effect of deletions of these potential N7 coordination sites is minor when compared to the more than 1000‐fold rate reduction observed for the G33c^7^G mutant (Table 1). Furthermore, the fact that the coordination of the ion to the scissile phosphate is solely observed in the product state suggests a more subtle role in phosphodiester cleavage. We speculate that a reason for this interaction in the product could be stabilization of a cyclophosphate conformation that hinders the reversible reaction (ligation).

By further revisiting the pre‐cleavage structure with respect to the exact location of this Mg^2+^ ion, we noticed that the O6 of G40 is also at a distance that would allow outershell interactions, and hence, may contribute to activation of G40 (the general base). Since this hypothesis has not been experimentally challenged in former studies,^31^, ^33^ we set out to synthesize a G40Ap mutant which lacks the O6, but otherwise provides the imine N1 and amine N2 (as encountered in the enol form of G; Figure 6 a). Qualitative HPLC cleavage assays indicated that the G40Ap mutant cleaved as fast as the wildtype ribozyme (Figure 6 b,c). Although the G40Ap mutant did not allow application of our former real‐time fluorescence assay for precise rate determination, because of interference with A57Ap, we analyzed the fluorescence response of the G40Ap mutant itself. We observed a decay in response to Mg^2+^ addition, with an apparent rate of 4.2 min^−1^, which was comparable to that of the wildtype ribozyme (Figure 6 e and Table 1). In contrast to G40Ap, the (O6 containing and N2 lacking) G40I mutant was significantly slower in cleaving (Figure 6 d), with a rate of 0.018 min^−1^ only (ssee Figure S6 and Table 1). This 220‐fold rate reduction provides evidence for a dominant role of the G40‐N2 functional group in TS recognition (as discussed above).

**Figure 6:**
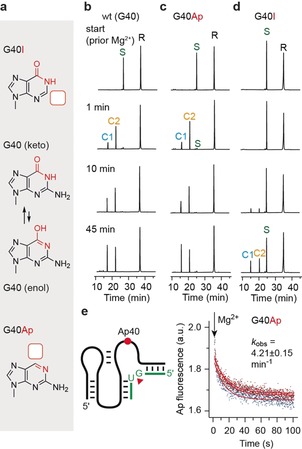
Atomic mutagenesis of G40 to analyze the impact of O6 versus N2 interactions in *env25* pistol ribozyme on self‐cleavage. a) Chemical structures of G40 in keto and enol forms and the corresponding nucleobase replacements (inosine and 2‐aminopurine) that lack either of the two exocyclic functionalities. b) Cleavage of the wildtype ribozyme analyzed by HPLC at the time points indicated. Reaction conditions: *c*(RNA)=55 μm RNA (1:1 ratio), 2 mm MgCl_2_, 100 mm KCl, 30 mm HEPES, pH 7.5, 23 °C. c) Same as (b) but for G40Ap. d) Same as (b) but for G40I. R: 47‐nt ribozyme, S: 11‐nt substrate, C1 and C2: 5‐nt and 6‐nt cleavage products. For HPLC conditions see Supporting Information. e) Fluorescence response of the G40Ap mutant during ribozyme cleavage. The single‐exponential fit of the obtained signal indicates a cleavage rate that is in agreement with the A57Ap assays (see Figure 5) and the HPLC assays (this Figure, panel b and c), confirming that the cleavage rates of G40Ap mutant and wild‐type ribozyme are comparable.

Another nucleotide, whose role in pistol ribozyme cleavage has been intensively debated, is G42.^18^, ^31^, ^33^ In the precatalytic structure, this guanine mediates the formation of a cleft (to accommodate the cleavage site), characterized by the hydrogen bond between the G42 N2 and the 2′O of ribose‐32 (2.7 Å), and by locating its O6 nearly equidistant to the G40 N2 (2.8 Å) and dG53 N2 (2.7 Å; see Figure S7a). In the TS analogue, G42 retains the interaction with ribose‐32 (2.4 Å) but simultaneously releases G53 and G40 (see Figure S7b). In the product structure, G42 is redirected towards G40 (2.8 Å distance between G42‐O6 and G40‐N2). Simultaneously, the cyclophosphate terminus of G53 slides away, with its N2 in over 4.4 Å distance to O6‐G42 (see Figure S7c). We underline that the strong link between the 2′‐OH of ribose‐32 and the exocyclic (Watson–Crick) NH_2_ of G42 is retained in all three structures (see Figure S7). The crucial role of the ribose‐32 2′OH is consistent with significantly reduced activities when this group was replaced by either H, OCH_3_, or NH_2_.^24^, ^31^, ^33^, ^37^

Our new structures of TS analogue and product significantly improve the mechanistic understanding of pistol ribozyme phosphodiester cleavage, for which we postulate the following scenario: In the precursor, a divalent ion (Mg^2+^) becomes innersphere‐coordinated to N7 of G33 and is additionally held in place by first shell water‐mediated hydrogen‐bond interactions, one to the 2′O of ribose‐32, and the other one to the *pro‐R* oxygen of the scissile phosphate (Figure 2 b). At the same time, G40 assists in proton transfer from the G53 2′O, which attacks the scissile phosphorus atom in‐line to the P−O5′ bond. In the TS, the nucleobase of G40 stabilizes the phosphorane in a bidendate manner (via N1⋅⋅⋅2′O and N2⋅⋅⋅*pro‐R* O), and simultaneously, the proximity of the divalent ion that remains innersphere‐coordinated to N7 of G33 assists in lowering the energy barrier by electrostatic interactions and by outershell coordination to the *pro‐R* nonbridging oxygen atom of the scissile phosphate (Figure 2 d). This process, in turn, places one of its first shell water molecules into appropriate distance for proton transfer (general acid) to the 5′O leaving group (Figure 2 d). The resulting cyclic phosphate product is embedded in a narrowed hydrogen‐bond network involving two divalent ions (Mg^2+^) in outersphere coordination to *pro‐R* and *pro‐S* oxygen centers, respectively (Figure 2 f). This arrangement may contribute to the stabilization of a cyclophosphate conformation that prevents ligation of the cleaved fragments (reversible reaction).

Only two other ribozymes have been structurally characterized by TS analogues so far. For the hairpin ribozyme, a vanadium oxide mimic was utilized for the first time.^28^ Direct interactions with nucleobase functional groups, which appeared to stabilize the electronic structure and geometry of the TS, had been revealed,^28^ as well as potentially involved water molecules.^29^ Comparable to pistol ribozyme, the superposition of pre‐cleavage, TS analogue, and product structures of hairpin ribozyme showed that the active site was essentially rigid, with motion confined to the scissile phosphate and the ribose pucker of the nucleotide upstream. Distinct from the pistol ribozyme, however, was that no divalent metal ion was present in the hairpin ribozyme active site and only nucleobases contributed to recognition of the cleavage site.

More recently, the crystal structure of a hammerhead ribozyme (HHRz) TS analogue was reported.^30^ This vanadate complex revealed significant rearrangements compared to the previously determined pre‐cleavage HHRz structures. The active site contracted, bringing a guanine (G10.1) closer to the cleavage site (Figure 7). This guanine resembles G33 in the pistol ribozyme, and similarly, it coordinated a divalent ion through N7 and a backbone phosphate (A9). This ion came closer to the scissile phosphate (3.9 Å). Although the distance is farther compared to the situation in pistol, the HHRz vanadate structure also suggested a contribution to TS stabilization through water‐mediated interaction with the scissile phosphate. A second divalent ion was observed to be innersphere coordinated to O6 of a guanine, which is considered the general base in the hammerhead ribozyme active site (G12). This metal ion likely helps tune the p*K*_a_ value of G12 to be appropriate for activation of the C17 2′O nucleophile. We also note that deletion of O6 in a G12Ap mutant of the hammerhead ribozyme caused a dramatic rate reduction (in the order of 10^3^),^38^ which reflects the significance of innersphere coordination of the metal ion to the G12 O6. In contrast, the second Mg^2+^ in the pistol ribozyme active site plays a minor role with respect to rate enhancement, but instead assists in conformational stabilization of the cyclic phosphate in the product complex.

**Figure 7:**
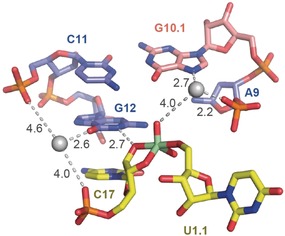
Hammerhead ribozyme active site (TS vanadate) reported by Mir et al., for purposes of comparison (PDB code: 5EAQ). Crucial atom distances indicating possible interactions are shown by dashed lines. The values in black represent distances in Å. For discussion see the main text.

In summary, our vanadate and product structures of the pistol ribozyme provide an unprecedented architectonic framework that sheds new light on this ribozyme's mechanism. A divalent hydrated metal ion that is innersphere coordinated to N7 of G33 in all three states (pre‐cleavage, transition state mimic, post‐cleavage) consolidates its major role in δ and β catalysis, and provides a rationale for the more than 1000‐fold loss in activity if its coordination to G33 is impaired. The cleavage is further supported by a second Mg^2+^ which stabilizes the 2′,3′‐cyclic phosphate in the product complex. The here provided structural framework may also stimulate further computational work on the pistol ribozyme mechanism.^39^

*Accession codes*. Protein Data Bank (PDB): atomic coordinates and structure factors have been deposited under the following accession codes: 6UEY for pistol ribozyme TS analog vanadate, 6UFJ for its 2′,3′ cyclophosphate product complex, 6UF1 for TS analog vanadate crystals soaked in Mn^2+^, and 6UFK for the 2′,3′ cyclophosphate product crystals soaked in Mn^2+^.

## Conflict of interest

The authors declare no conflict of interest.

## Supporting information

As a service to our authors and readers, this journal provides supporting information supplied by the authors. Such materials are peer reviewed and may be re‐organized for online delivery, but are not copy‐edited or typeset. Technical support issues arising from supporting information (other than missing files) should be addressed to the authors.

Supplementary
