# Supplementary material for: Crucial Roles of Two Hydrated Mg2+ Ions in Reaction Catalysis of the Pistol Ribozyme
Source: Angew Chem Int Ed Engl. 2020 Jan 9;59(7):2837–43. doi: 10.1002/anie.201912522 (PMC7027511; doi:10.1002/anie.201912522)
Supplement: Supplementary file 1 — Supplementary [file ANIE-59-2837-s001.pdf]

Angewandte Chemie
Eine Zeitschrift der Gesellschaft Deutscher Chemiker

Supporting Information

Crucial Roles of Two Hydrated Mg^{2+} Ions in Reaction Catalysis of the Pistol Ribozyme

Marianna Teplova[+], Christoph Falschlunger[+], Olga Krasheninina[+], Michaela Egger, Aiming Ren, Dinshaw J. Patel, and Ronald Micura**

anie_201912522_sm_miscellaneous_information.pdf

Supporting Information

Contents

Methods **S2**

RNA solid-phase synthesis, deprotection, and purification S2
Mass spectrometry S3
Crystallization and structure determination S3
Ribozyme cleavage (HPLC assay) S4
Kinetics of ribozyme cleavage (2-aminopurine fluorescence assay) S4

Supporting Figures

Supporting Figure S1 S5
Supporting Figure S2 S6
Supporting Figure S3 S7
Supporting Figure S4 S8
Supporting Figure S5 S9
Supporting Figure S6 S10
Supporting Figure S7 S11

Supporting Tables

Supporting Table S1 S12
Supporting Table S2 S13

References **S14**

Methods

RNA solid-phase synthesis, deprotection, and purification

All RNAs were assembled on an ABI392 synthesizer at 1 µmol scale using 2'-*O*-TOM nucleoside phosphoramidites (ChemGenes), polystyrene supports (GE Healthcare, Custom Primer Support^{TM}, 80 µmol g^{-1}; PS 200 and Primer Support^{TM} 5G, 300 µmol g^{-1}) and CPG supports (ChemGenes, CPG 1000 Å, 30-40 µmol g^{-1}).

Standard RNA synthesis cycle: 1) detritylation with dichloroacetic acid/1,2-dichloroethane (4/96) (120 s); 2) coupling with phosphoramidites in acetonitrile (0.1 M) and benzylthiotetrazole in acetonitrile (0.3 M) (180 s); 3) capping with Cap A: phenoxyacetic anhydride (0.2 M) in dry THF and Cap B: *N*-methyl imidazole and *sym*-collidine (0.2 M each) in dry THF (2 x 15 s, Cap A/Cap B, 1:1); 4) oxidation with iodine (20 mM) in tetrahydrofuran (THF)/pyridine/H_2O (40/1/9) (60 s).

Commercially available modified nucleoside building blocks of 2-aminopurine riboside, inosine, and 7-deazaadenosine (ChemGenes or Glen Research) were incorporated using modified synthesis cycles with longer coupling times (up to 6 min).

Base deprotection and cleavage of oligoribonucleotides from the support were carried out by treatment with a mixture of 40% aqueous methylamine and 30% aqueous ammonia (600 µL, 1:1 v/v) in a screw-cap vial at 65 °C for 15 minutes. The resulting solution was filtered off, the support was washed with a mixture of acetonitrile, methanol and water (300 µL, 1:1:1 v/v) twice, and then combined filtrates were evaporated to dryness in a SpeedVac concentrator.

The 2'-*O*-TOM protecting groups were removed by incubation of oligoribonucleotides in a mixture of *N*-methyl-2-pyrrolidone, trimethylamine, triethylamine trihydrofluoride (200 µL, 1:1:1 v/v) at 65 °C for 2 h. The reaction was cooled in freezer briefly, and then 25 µL of 3 M sodium acetate was added followed by ~1 mL of 1-butanol. The mixture was cooled at -20 °C for 1 h. The formed precipitate of the oligonucleotide sodium salts was centrifuged (15 min, 14,000 rpm, 4 °C), washed with ethanol or acetone and air-dried.

Quality assessment of the crude RNAs was performed using anion-exchange HPLC on a Dionex DNAPac PA-100 column (4 x 250 mm); conditions: flow rate 1 mL min^{-1}; eluent A: 25 mM Tris-HCl, pH 8.0, 6 M urea, eluent B: 500 mM $NaClO_4$, 25 mM Tris-HCl, pH 8.0, 6 M urea; gradient: 0 - 60% B in 45 minutes; 60 °C, UV detection at 260 nm.

The desired oligoribonucleotides were isolated by semi-preparative anion-exchange HPLC on a Dionex DNAPac PA-100 column (9 x 250 mm); Conditions: flow rate 2 mL min^{-1}; see above.

The product fractions were desalted using C18 Sep-Pak cartridges (Waters Corporation). The quality of the product was analyzed by anion-exchange HPLC and reversed-phase LC-ESI-MS. Sequences and MS data for all the obtained RNAs are shown in Supporting Table S2.

Mass spectrometry

All experiments were performed on a Finnigan LCQ Advantage MAX ion trap instrumentation connected to a Thermo Fisher Ultimate 3000 HPLC system. RNAs were analyzed in the negative-ion mode with a potential of −4 kV applied to the spray needle. LC: Sample (200 pmol RNA dissolved in 30 µL of 20 mM ethylenediamine tetraacetic acid (EDTA) solution; average injection volume: 30 µL); column (Waters XTerraMS, C18, 2.5 µm; 1.0 × 50 mm) at 21°C; flow rate: 0.1 mL/min; eluant A: 8.6 mM triethylamine (TEA), 100 mM 1,1,1,3,3,3-hexafluoroisopropanol in H_2O (pH 8.0); eluant B: methanol; gradient: 0–100% B in A within 30 min; UV detection at 254 nm.

Crystallization and structure determination

The sample for crystallization of the transition state vanadate complex was generated by annealing the three purified oligonucleotide strands (Supporting Figure S1c) at a concentration of 0.22 mM and 70 °C for 3 min in a buffer containing 10 mM K-cacodylate, pH 6.0, 50 mM KCl, 10 mM $MgCl_2$, and 5 mM NH_4VO_3 followed by incubation at room temperature for 5 min and then cooling on ice for 30 min prior to setting up crystallization trials. The crystals of the tri-stranded pistol ribozyme construct were grown at 20 °C over a period of 2 weeks using the hanging-drop vapor diffusion method by mixing 1µl of RNA sample with equal volume of reservoir solution containing 0.1 M Na-acetate, pH 4.6, 0.02 M $CaCl_2$, 25% MPD and 4.5 mM NH_4VO_3. The crystals were transferred into the cryostabilization solution containing 0.1 M Na-Hepes, pH 7.5, 0.05 M $MgCl_2$, 35% MPD, and 0.1 M NH_4VO_3 and incubated at 20 °C for 3 hours. The ammonium metavanadate solution was freshly prepared as 0.2 M stock in 10 mM Na-cacodylate pH 7.4, heated to 95 °C until dissolved and titrated with 1M NaOH to pH 9.0. For data collection, crystals were flash-frozen in liquid nitrogen.

The sample for crystallization of the 2',3'-cyclophosphate product was generated be annealing the two purified oligonucleotide strands (Supporting Figure S1d) at a concentration of 0.25 mM at 70 °C for 3 min in a buffer containing 10 mM K-cacodylate, pH 6.0, 50 mM KCl, and 10 mM $MgCl_2$ followed by incubation at room temperature for 5 min and then cooling on ice for 30 min prior to setting up crystallization trials. The crystals were grown at 20 °C over a period of 2-4 weeks using the hanging-drop vapor diffusion method by mixing 1µl of RNA sample with equal volume of reservoir solution containing 0.05 M Na-Hepes, pH 7.5, 0.1 M $MgCl_2$, and 25-30% MPD. The crystals were cryostabilized in crystallization solution supplemented with 35% MPD and flash-frozen in liquid nitrogen for data collection.

For Mn2+ soaking experiments, crystals were gradually transferred into the cryostabilization solution containing 0.05 M Na-Hepes, pH 7.5, 0.1 M $MnCl_2$ and 35% MPD at 20 °C and incubated for 20 h.

X-ray diffraction data were collected on beamline 24ID at the Advanced Photon Source (APS). The diffraction data on the vanadate complex and the 2',3'-cyclophosphate product crystals were processed using HKL2000 (HKL Research). The diffraction data on Mn2+-soaked crystals were integrated and scaled using on-site RAPD automated programs (https://rapd.nec.aps.anl.gov/rapd/). Initial phases were obtained by molecular replacement with previously reported structure of *env27* pistol ribozyme (PDB code: 5KTJ) as the search model using Phaser (ref 1) in Phenix suite (ref 2). Iterative model building and refinement was performed by using COOT (ref 3) and Phenix package (ref 2). Crystal diffraction data and refinement statistics are shown in Supporting Table S1.

Ribozyme cleavage (HPLC assay)

Nanomole aliquots of the ribozyme and substrate strands were diluted with reaction buffer to final concentrations: 55 µM of each RNA strand, 30 mM HEPES, pH 7.5, 100 mM KCl. Cleavage reaction was initiated by the addition of $MgCl_2$ solution to reach a final concentration of 2 mM. Samples (4 µL) were drawn after the indicated time points, quenched with the addition of equal amounts of 40 mM EDTA solution, and then diluted with water to 100 µL. For the determination of HPLC reaction kinetics of fast cleaving ribozyme mutants (A37c^7A, A38c^7A, A37c^7A– A38c^7A) the stop solution was 40 mM EDTA, 6 M urea (10 µL) applied at 80 °C, and followed by dilution with 100 µL of water. For slow cleaving ribozyme mutants, only 4 µM of stop solution applied at room temperature was used. Reaction progress was monitored by anion-exchange HPLC as described above.
We assayed the ribozymes under single turnover conditions for reasons of comparison to our previous studies on twister, pistol, and twister-sister ribozymes (*Nat. Commun*. 2014, 5, 5534; *Nat. Chem. Biol*. 2016, 12, 709; *Nat. Commun*. 2017, 8, 1180; PNAS 2019, 116, 10783) that were analysed in 1:1 ratio of the two strands.

Kinetics of ribozyme cleavage (2-aminopurine fluorescence assay)

Observed rates k_{obs} were obtained by following the fluorescence change of the Ap labeled ribozyme complex over time and fitting a single-exponential equation to the data. Data were collected on a Cary Eclipse spectrometer (Varian, Australia) equipped with a peltier block, and a RX2000 stopped-flow apparatus (Applied Photophysics Ltd., UK) using the following parameters: excitation at 308 nm, detection at 372 nm; data point collection increments 0.2 s; slit widths 10 nm.

Stock solutions were prepared for each Ap labeled two-stranded ribozyme complex (1.0 µM of ribozyme complex (1:1 ratio) in 50 mM KMOPS pH 7.5, 100 mM KCl) and for $MgCl_2$ (20 mM of $MgCl_2$ in 50 mM KMOPS pH 7.5, 100 mM KCl). The RNA samples were heated to 90 °C for 2 min, and then allowed to cool slowly to room temperature. Mixing equal volumes of the stock solutions *via* the stopped-flow apparatus resulted in a final concentration of 0.5 µM for a ribozyme complex and of 10 mM for $MgCl_2$.

The stopped-flow fluorescence data were fitted using a three-parameter (A_1, A_2 and k_{obs}) single-exponential equation for 1:1 stoichiometry: $F(t) = A_1 + A_2 \cdot \exp(-k_{obs} \cdot t)$, where A_1 is final fluorescence, and $A_2 \cdot \exp(-k_{obs} \cdot t)$ is change in fluorescence over time (t) at the observed rate k_{obs}.

The stated values (Table 1) are arithmetic means from at least three experiments. The software package OriginPro 2018 (OriginLab, USA) was used to process and illustrate the data.

a

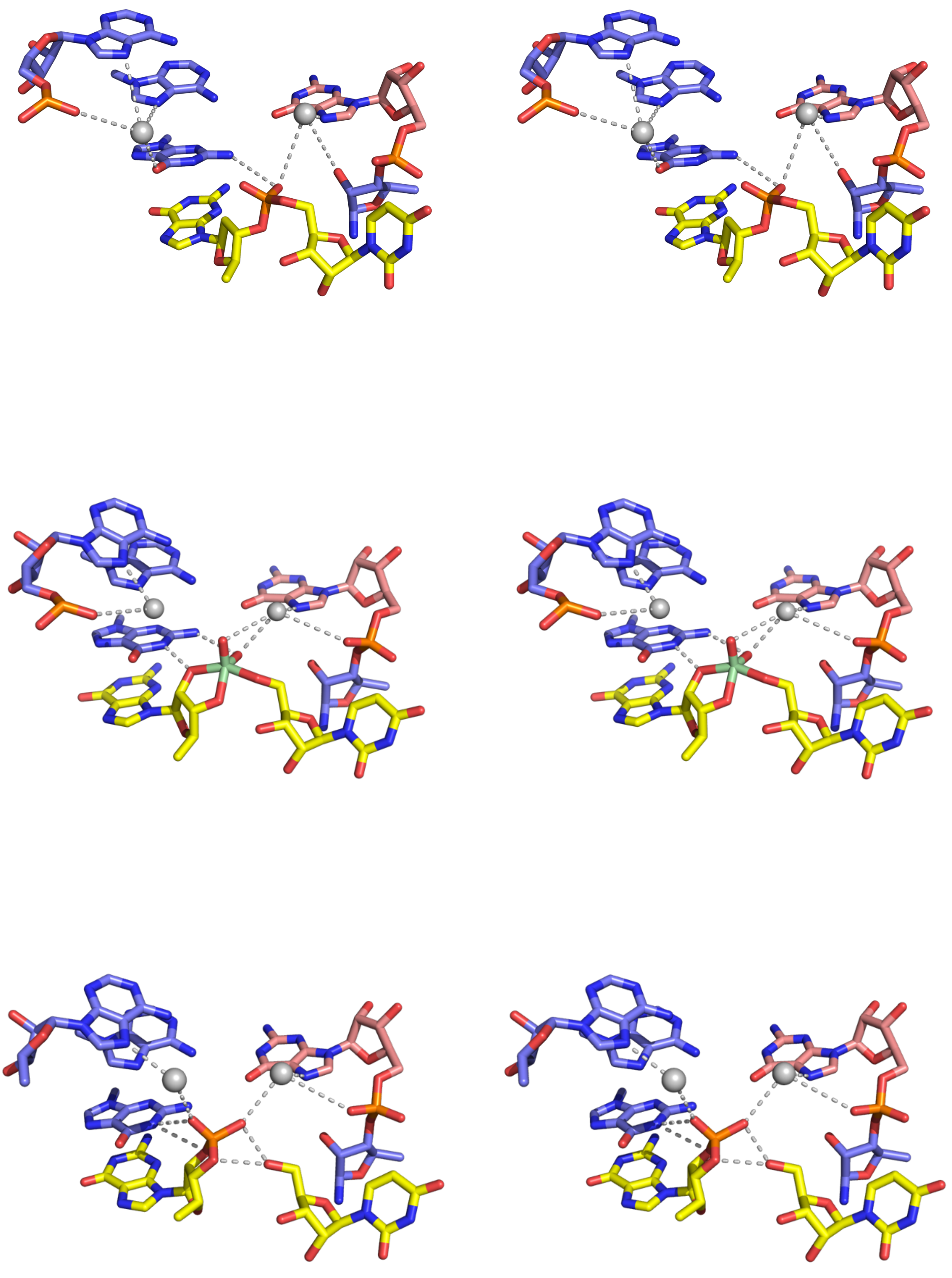

b

c

Supporting Figure S1. Crystal structures for pistol ribozyme pre-cleavage (a), transition state analog (b), and post-cleavage (c) conformations; active sites in wall-eyed stereo mode (PyMol). Crucial atom distances (below 4.5 Å) that indicate direct or water-mediated hydrogen bonds and/or metal ion interactions are illustrated by dashed lines. Note that the number of indicated distances can exceed the number of possible hydrogen bonds for a particular atom. The views correspond to Figure 2 in the main text. For assignments and discussion see the main text.

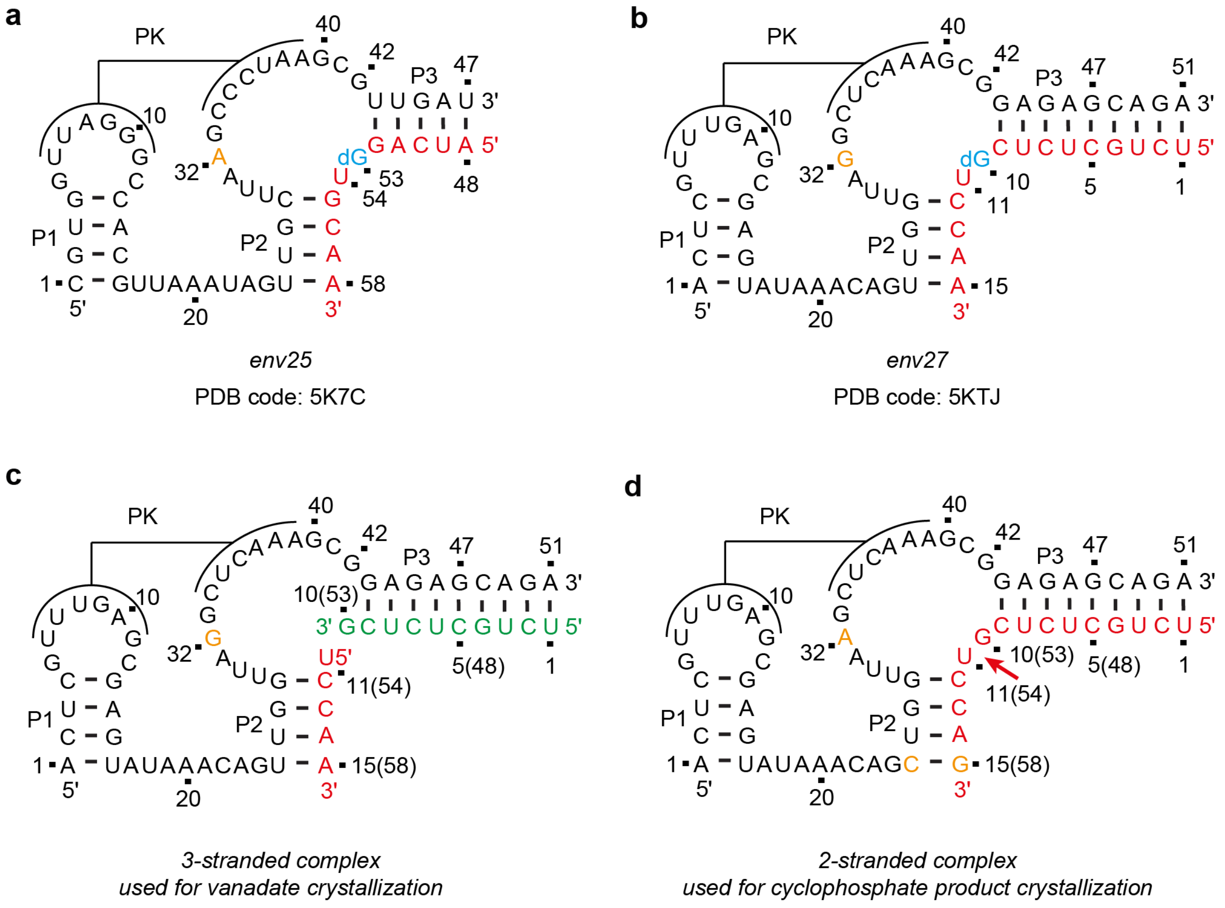

Supporting Figure S2. Pistol ribozyme RNA design used for crystallization experiments. (**a**) Bimolecular RNA complex used for crystallization to obtain the pre-cleavage structure by Ren, A. et al. (2016) *Nature Chemical Biology*, 12(9), pp.702–708; (**b**) Bimolecular RNA complex used for crystallization to obtain the pre-cleavage structure by Nguyen, L.A. et al. (2017) *Proc. Natl. Acad. Sci. USA*, 114(5), pp.1021–1026; (**c**) Trimolecular RNA complex used for crystallization to obtain the transition state analog structure of *this work*; (**d**) Bimolecular RNA complex used for crystallization to obtain the 2',3'-cyclophosphate structure of *this work*; red arrow indicates cleavage during crystallization. General color code: ribozyme strand (1st strand, black), substrate strand (2nd strand, red), substrate strand fragments (2nd strand red, 3rd strand green), modifications are shown in cyan, selected sequence alterations are highlighted in orange; pseudoknot (PK).

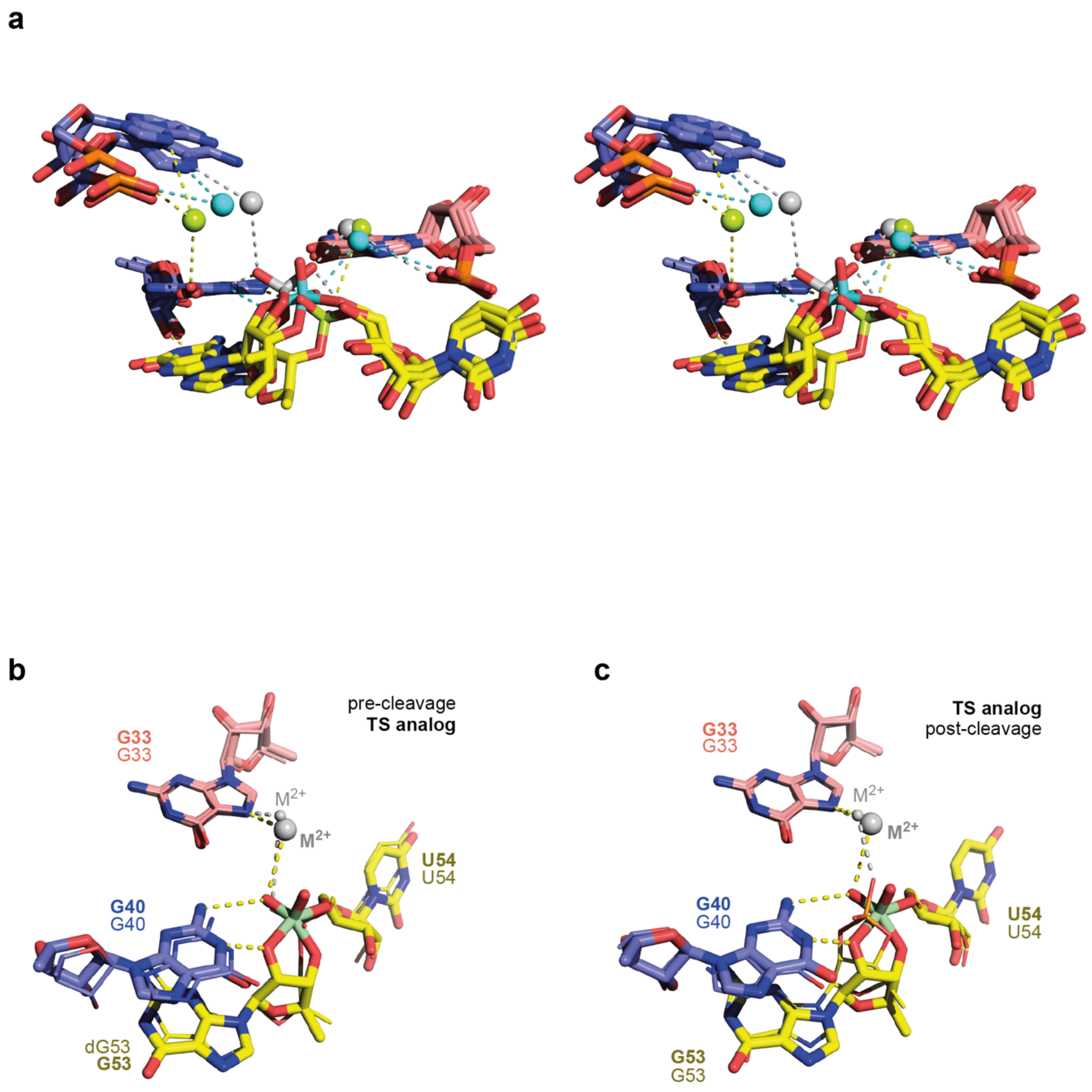

Supporting Figure S3. Superpositions of pistol ribozyme states to visualize the conformational rearrangements during the transesterification reaction. **a**) Stereo view of the active site of the pistol ribozyme in superpositions of pre-cleavage (pdb 5K7C), vanadate complex (this work), and post-cleavage structures (this work). Wall-eyed stereo mode in PyMol was used; **b**) Superposition of transition-state (TS) analog (stick presentation) with pre-cleavage structures (thin line presentation); **c**) Superposition of transition-state (TS) analog (stick presentation) with post-cleavage structures (thin line presentation). For discussion, see main text.

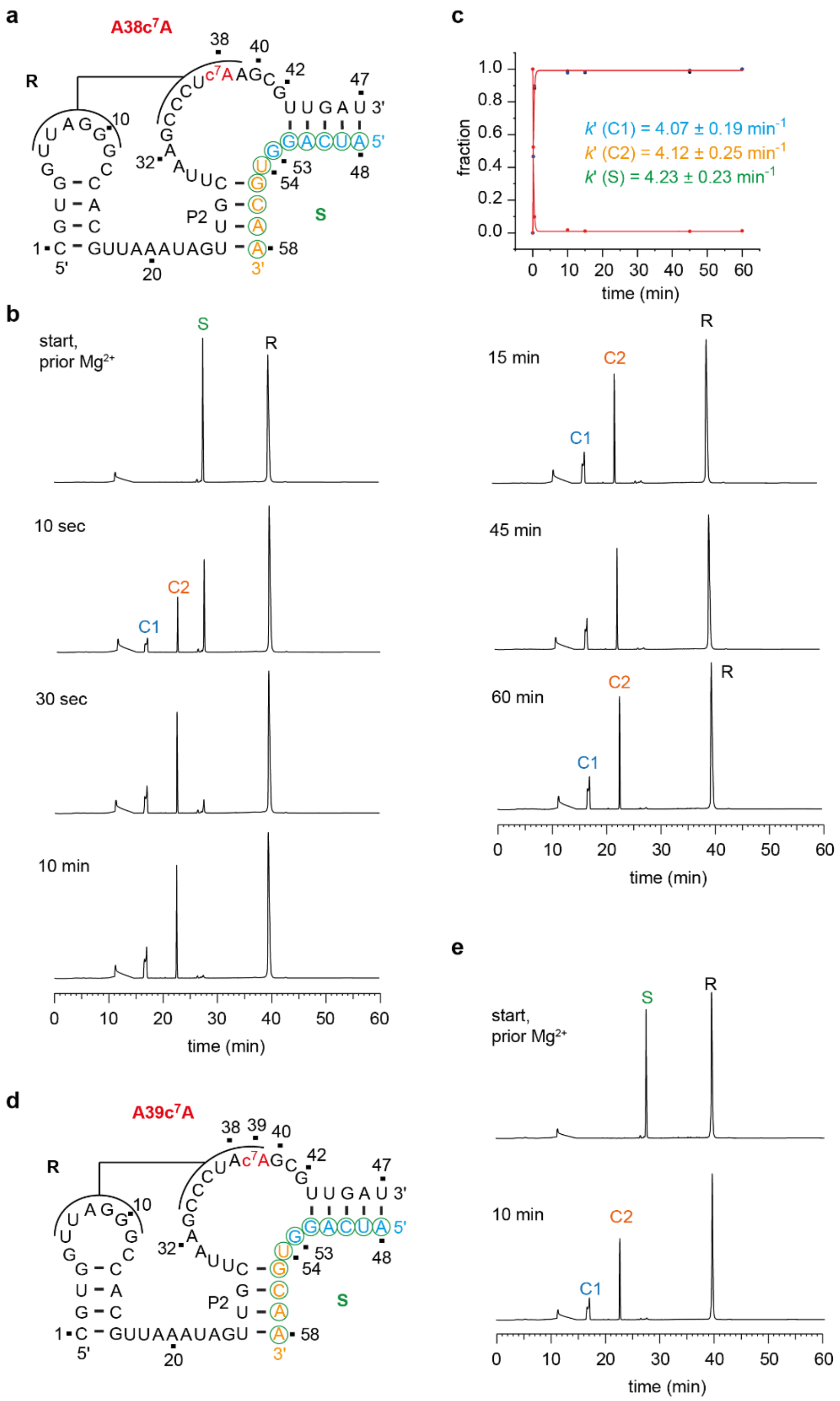

Supporting Figure S4. Self-cleavage of A38c^{7}A and A39c^{7}A *env25* pistol ribozyme mutants. **a**) A38c^{7}A modified RNA set-up; **b**) Anion exchange HPLC traces of the reaction time course; reaction conditions: c(RNA) = 55 µM each RNA strand (1:1 ratio); 2 mM $MgCl_2$, 100 mM KCl, 30 mM HEPES, pH 7.5, 23 °C. The reaction was stopped at the indicated time points by drawing a 4 µL sample and mixing it with 10 µL of stop solution (40 mM Na_2EDTA, 6 M urea, 80°C), followed by dilution into 100 µL of water. HPLC conditions: Dionex DNAPac column (4x250 mm), 60 °C, 1 ml min^{-1}, 0–60% buffer B in 45 min. Buffer A: Tris–HCl (25 mM), urea (6 M), pH 8.0. Buffer B: Tris–HCl (25 mM), urea (6 M), $NaClO_4$ (0.5 M), pH 8.0. Note that the fragment C1 appears as broad double peak due to overlapping elution with the ingredients of the stop solution (see also absorbance at 11 to 13 min); **c**) Estimation of observed rate from fractions (S, C1, and C2) obtained by HPLC analysis. **d**) A39c^{7}A modified RNA set-up; **e**) Same as (b) but for A39c^{7}A mutant.

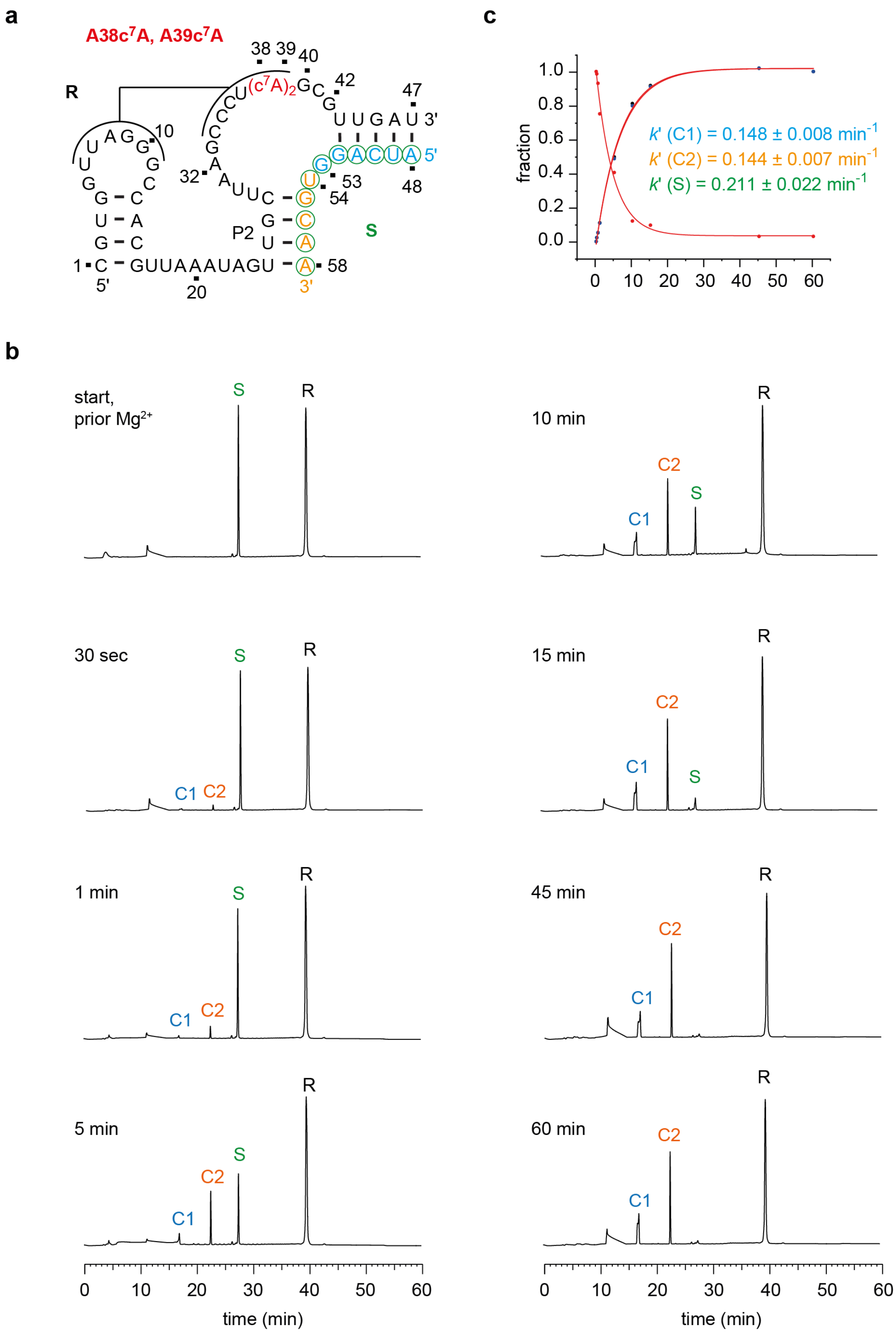

Supporting Figure S5. Self-cleavage of the A38c^{7}A–A39c^{7}A *env25* pistol ribozyme mutant. **a**) A38c^{7}A–A39c^{7}A modified RNA set-up; **b**) Anion exchange HPLC traces of the reaction time course; reaction conditions: c(RNA) = 55 µM each RNA strand (1:1 ratio); 2 mM $MgCl_2$, 100 mM KCl, 30 mM HEPES, pH 7.5, 23 °C. The reaction was stopped at the indicated time points by drawing a 4 µL sample and mixing it with 10 µL of stop solution (40 mM Na_2EDTA, 6 M urea, 80°C), followed by dilution into 100 µL of water. HPLC conditions: Dionex DNAPac column (4x250 mm), 60 °C, 1 ml min^{-1}, 0–60% buffer B in 45 min. Buffer A: Tris–HCl (25 mM), urea (6 M), pH 8.0. Buffer B: Tris–HCl (25 mM), urea (6 M), $NaClO_4$ (0.5 M), pH 8.0. Note that the fragment C1 appears as broad double peak due to overlapping elution with the ingredients of the stop solution (see also absorbance at 11 to 13 min); **c**) Estimation of observed rate from fractions (S, C1, C2) obtained by HPLC analysis.

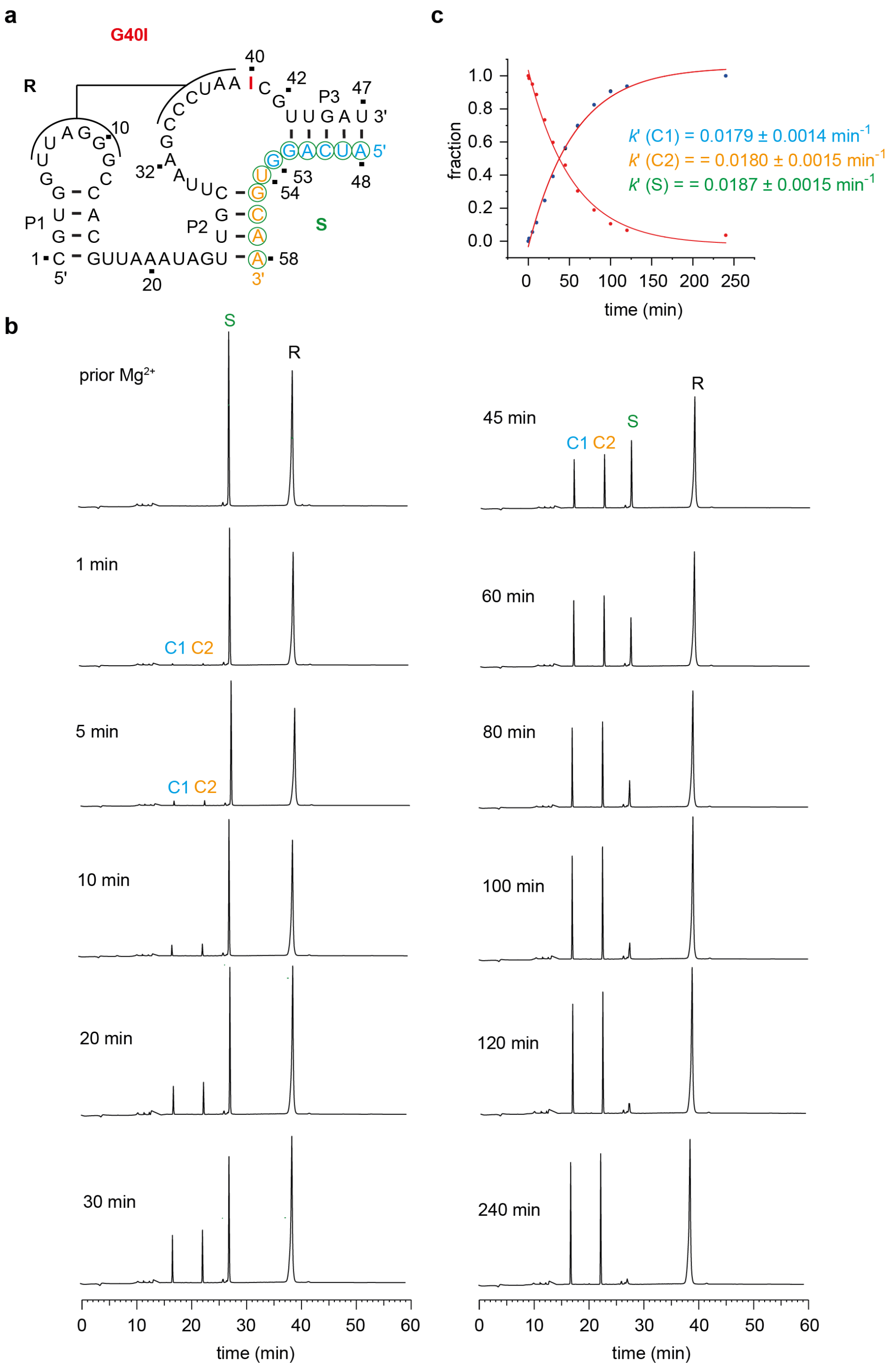

Supporting Figure S6. Self-cleavage of the G40I *env25* pistol ribozyme mutant. **a**) G40I modified RNA set-up; **b**) Anion exchange HPLC traces of the reaction time course; reaction conditions: c(RNA) = 55 µM each RNA strand (1:1 ratio); 2 mM $MgCl_2$, 100 mM KCl, 30 mM HEPES, pH 7.5, 23 °C. The reaction was stopped at the indicated time points by drawing a 4 µL sample and mixing it with 4 µL of stop solution (40 mM Na_2EDTA, 23°C), followed by dilution into 100 µL of water. HPLC conditions: Dionex DNAPac column (4x250 mm), 60 °C, 1 ml min^{-1}, 0–60% buffer B in 45 min. Buffer A: Tris–HCl (25 mM), urea (6 M), pH 8.0. Buffer B: Tris–HCl (25 mM), urea (6 M), $NaClO_4$ (0.5 M), pH 8.0); **c**) Estimation of observed rate from fractions (S, C1, C2) obtained by HPLC analysis.

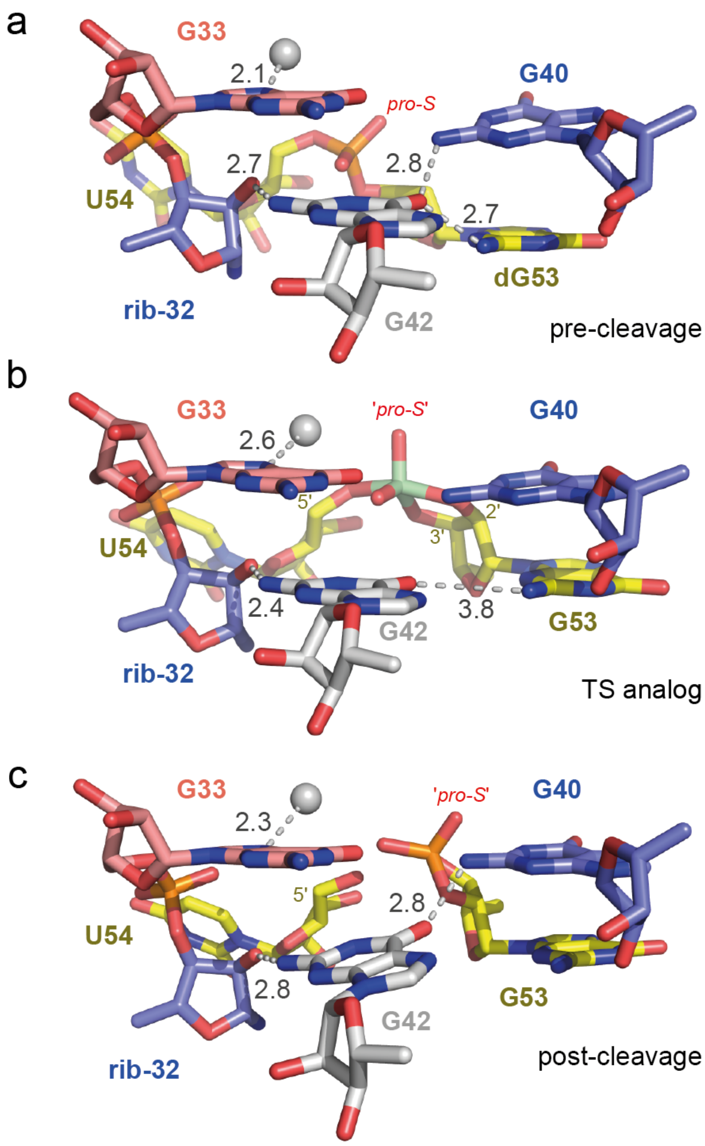

Supporting Figure S7. Structural rearrangements of G42 in the ribose-32–G42–G40 cleft that accommodate the G53–U54 cleavage site in the pistol ribozyme illustrated for pre-cleavage (a), transition state analog (b), and post-cleavage (c) conformations. Crucial atom distances indicating possible hydrogen bonds are shown by dashed lines; the values in black represent distances in Å. For discussion see the main text.

Supporting Table S1. X-ray diffraction and refinement statistics for pistol ribozyme vanadate and 2',3'-cyclophosphate product complexes

Crystal	Vanadate complex	Vanadate complex Mn^{2+} soak	2',3'-cyclophosphate	2',3'-cyclophosphate Mn^{2+} soak
**Data Collection**	24-ID-E	24-ID-C	24-ID-C	24-ID-C
Space group	P42 21 2	P42 21 2	P42 21 2	P42 21 2
Cell dimensions				
a,b,c (Å)	91.8 91.8 123.4	90.6 90.6 122.6	90.7 90.7 121.7	90.4 90.4 121.7
a,b,c (°)	90.0 90.0 90.0	90.0 90.0 90.0	90.0 90.0 90.0	90.0 90.0 90.0
Wavelength (Å)	0.97918	1.89170	0.97918	1.89170
Resolution (Å)	45.9 - 2.8 (2.85 - 2.80)	122.6 - 2.90 (3.08 - 2.90)	50.0 - 2.65 (2.70 - 2.65)	90.4 - 2.97 (3.15 - 2.97)
R_{pim}	0.017 (0.26)	0.046 (5.3)	0.015 (0.28)	0.052 (33.1)
$I/\sigma I$	37.9 (2.9)	11.2 (0.2)	43.1 (1.7)	11.3 (0.2)
Completeness (%)	99.8 (99.9)	98.9 (94.4)	99.8 (97.4)	99.2 (97.7)
Redundancy	10.2 (10.0)	8.5 (8.8)	11.2 (8.9)	5.9 (6.0)
**Refinement**				
Resolution (Å)	20.0-2.8	20.0-3.1	40.0-2.65	20.0-3.2
No. reflections	13303	17411	15194	15425
R_{work}/R_{free}	21.1/25.1	19.9/25.5	19.0/23.9	21.1/25.7
No. atoms				
RNA	2764	2764	2764	2764
Cations	14	17	17	22
B-factors (Å^2)				
RNA	120	118	92	134
Cations	98	137	87	144
R.m.s. deviations				
Bond length (Å)	0.05	0.06	0.05	0.05
Bond angles (°)	1.2	1.2	1.1	1.1

Supporting Table S2. Components of pistol ribozyme studied in the work.

RNAs	*Sequence* [a]	*Molecular weight*	
		Found[b] *m/z*	Calcd *m/z*
3-Stranded complex used for vanadate crystallization (Supporting Figure S2,c)			
R, 51-nt	5'-ACUCGUUUGAGCGAGUAUAAACAGUUGG UUAGGCUCAAAGCGGAGAGCAGA-3'	16538.86	16538.05
C1, 5-nt	5'-UCCAA-3'	1512.93	1512.99
C2, 10-nt	5'-UCUGCUCUCG-3'	3073.74	3073.86
2-Stranded complex used for cyclophosphate product crystallization (Supporting Figure S2,d)			
R', 51-nt	5'-ACUCGUUUGAGCGAGUAUAAACAGCUGG UUAAGCUCAAAGCGGAGAGCAGA-3'	16522.13	16521.07
S', 15-nt	5'-UCUGCUCUCGUCCAG-3'	4665.12	4664.82
2-Stranded complex used for cleavage assays			
Ribozyme variants			
G40 (wild-type)	5'-CGUGGUUAGGGCCACGUUAAAUAGUU GCUUAAGCCCUAAGCGUUGAU-3'	15080.43	15080.07
A38c[7]A	5'-CGUGGUUAGGGCCACGUUAAAUAGUU GCUUAAGCCCU*c[7]A*AGCGUUGAU-3'	15079.30	15078.08
A39c[7]A	5'-CGUGGUUAGGGCCACGUUAAAUAGUU GCUUAAGCCCUA*c[7]A*GCGUUGAU-3'	15080.87	15078.08
A38c[7]A/A39c[7]A	5'-CGUGGUUAGGGCCACGUUAAAUAGUU GCUUAAGCCCU*c[7]Ac[7]A*GCGUUGAU-3'	15077.59	15076.09
G40Ap	5'-CGUGGUUAGGGCCACGUUAAAUAGUU GCUUAAGCCCUAA*2Ap*CGUUGAU-3'	15065.40	15064.07
G40I	5'-CGUGGUUAGGGCCACGUUAAAUAGUU GCUUAAGCCCUAA*I*CGUUGAU-3'	15065.19	15065.06
Substrate variants			
A57 (wild-type)	5'-AUCAGGUGCAA-3'	3513.51	3513.20
A57Ap	5'-AUCAGGUGC*2Ap*A-3'	3513.47	3513.20

[a] *c[7]A* – 7-deazaadenosine, *2Ap* – 2-aminopurine riboside, *I* – inosine. [b] Reversed-phase LC-ESI mass spectrometry (see Methods).

References

1. McCoy, A.J., Grosse-Kunstleve, R.W., Adams, P.D., Winn, M.D., Storoni, L.C., & Read, R.J. Phaser crystallographic software. *Journal of Applied Crystallography* **40**, 658-674 (2007).

2. Adams, P.D. et al. PHENIX: a comprehensive Python-based system for macromolecular structure solution. *Acta Crystallographica Section D: Biological Crystallography* **66**, 213-221 (2010).

3. Emsley, P., Lohkamp, B., Scott, W.G. & Cowtan, K. Features and development of Coot. *Acta Crystallographica Section D: Biological Crystallography* **66**, 486-501 (2010).
